# Characterization of a novel class A carbapenemase PAD-1 from *Paramesorhizobium desertii* A-3-E^T^, a strain highly resistant to β-lactam antibiotics

**DOI:** 10.1038/s41598-017-07841-1

**Published:** 2017-08-21

**Authors:** Ruichen Lv, Jingyu Guo, YanFeng Yan, Rong Chen, Lisheng Xiao, Min Wang, Nan Fang, Chengxiang Fang, Yujun Cui, Ruifu Yang, Yajun Song

**Affiliations:** 1 State Key Laboratory of Pathogen and Biosecurity, Beijing Institute of Microbiology and Epidemiology, Beijing, 100071 China; 2 College of Life Sciences, Wuhan University, Wuhan, 430072 China

## Abstract

Although clinical antibiotic-resistant bacteria have attracted tremendous attention in the microbiology community, the resistant bacteria that persist in natural environments have been overlooked for a longtime. We previously proposed a new species *Paramesorhizobium desertii*, isolated from the soil of the Taklimakan Desert in China that is highly resistant to most β-lactam antibiotics. To identify potential β-lactamase(s) in this bacteria, we first confirmed the carbapenemase activity in the freeze–thawed supernatant of a *P*. *desertii* A-3-E^T^ culture using the modified Hodge assay. We then identified a novel chromosome-encoded carbapenemase (PAD-1) in strain A-3-E^T^, using a shotgun proteomic analysis of the supernatant and genomic information. The bioinformatics analysis indicated that PAD-1 is a class A carbapenemase. Subsequent enzyme kinetic assays with purified PAD-1 confirmed its carbapenemase activity, which is similar to that of clinically significant class A carbapenemases, including BKC-1 and KPC-2. Because the location in which A-3-E^T^ was isolated is not affected by human activity, PAD-1 is unlikely to be associated with the selection pressures exerted by modern antibiotics. This study confirmed the diversity of antibiotic-resistant determinants in the environmental resistome.

## Introduction

Antibiotics remain one of the most important weapons with which human can combat infectious diseases. However, antibiotic-resistance genes have emerged and spread in both pathogenic and non-pathogenic bacteria worldwide, with breath-taking speed and unprecedented coverage. Various resistance mechanisms have been developed by different bacteria, against almost all commercially available antibiotics^1^.

Carbapenems have a very broad spectrum of activities against many Gram-negative bacteria, and are the very first-line therapy for the treatment of clinical infections caused by the Enterobacteriaceae that produce extended-spectrum β-lactamases^2^. However, the effectiveness of carbapenems is now challenged by the increasing number of carbapenemases identified in clinical strains in recent years^3, 4^. Carbapenemases are a group of β-lactamases that can hydrolyse carbapenems. Based on their protein sequence homologies, β-lactamases are classified into four molecular classes, A, B, C, and D, and carbapenemases are found in classes A, B, and D^5^.

Since the first class A carbapenemase was reported in 1991 in *Serratia marcescens* ^6^, it has become one of the most important carbapenemases in clinical microbiology. Class A carbapenemases can be divided phylogenetically into six different groups: GES, KPC, SME, IMI/NMC-A, SHV-38, and SFC-12^3^. The genes encoding the class A carbapenemases can be plasmid-borne or located on the chromosome of the host bacterium. For instance, the *bla*_*GES*_ genes usually occur as gene cassettes on class I integrons in the chromosome of *Pseudomonas aeruginosa* ^7^, whereas the *bla*_*KPC*_ genes are normally flanked by transposable elements on plasmids in *Klebsiella pneumoniae* ^8^. With the aid of their flanking mobile elements (integrons or transposons), genes encoding class A carbapenemases are susceptible to dissemination among different bacteria.

In our recent study, a new species of a novel genus, designated *Paramesorhizobium desertii*, was isolated from Taklimakan Desert soil samples, and shown to be highly resistant to most β-lactam antibiotics^9^. Here, we report the identification of a novel chromosome-encoded class A carbapenemase from the type strain of this species, A-3-E^T^, and the enzyme kinetic parameters of this carbapenemase.

## Results

### Identification of PAD-1

**Figure 1:**
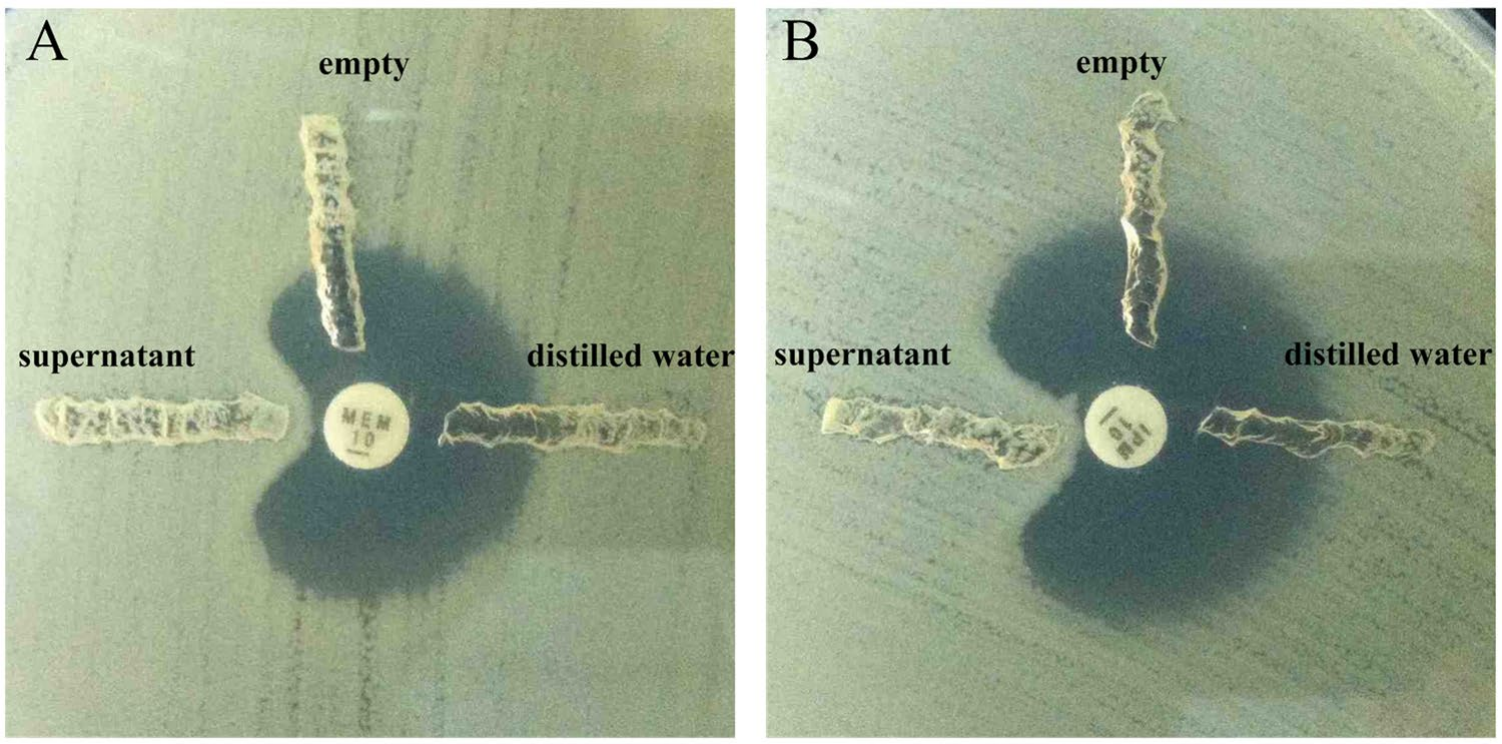
Modified Hodge assay confirming β-carbapenemase activity in the freeze-thawed supernatant of A-3-E^T^. Carbapenemase in the A-3-E^T^ supernatant hydrolysed carbapenems and distorted the inhibition zones. (**A**) MH plate with meropenem disc; (**B**) MH plate with imipenem disc.

Figure 1 shows the results of a modified Hodge test for carbapenemase activity. On Mueller-Hinton (MH) plates containing meropenem or imipenem discs, carbapenem-sensitive *Escherichia coli* ATCC 25922 grew well along the grooves containing the A-3-E^T^ supernatant, whereas it did not grow along the empty groove or the groove containing distilled water. This suggests that the carbapenemase-resistant phenotype of A-3-E^T^ is unlikely to be associated with drug efflux or other mechanisms, but that the carbapenemase is present in the cell culture supernatant.

A subsequent shotgun proteomic analysis identified 20 proteins in the freeze-thawed supernatant (Table 1), among which ATN84_21655 was the only one in the A-3-E^T^ genome to be annotated as a β-lactamase^10^. We designated it PAD-1, according to its species name $\underline{{\boldsymbol{Pa}}}ramesorhizobium\,\underline{{\boldsymbol{d}}}esertii$ ^9^.

**Table 1:** Supernatant proteins identified with a proteomic analysis.

Gene ID	MW (Da)	Peptides matched	Score	Gene annotation
ATN84_17990	26,143	58	777	hypothetical protein
ATN84_21655	31,752	17	713	class A beta-lactamase
ATN84_20110	57,004	10	639	hypothetical protein
ATN84_18000	24,800	44	634	hypothetical protein
ATN84_24700	433,47	13	502	4-hydroxyphenylpyruvate dioxygenase
ATN84_05690	130,058	17	460	thioredoxin
ATN84_09425	18,568	7	458	peptidyl prolylisomerase
ATN84_02110	57,439	7	356	chaperonin GroEL
ATN84_18040	14,305	6	277	host cell attachment protein
ATN84_08840	12,176	4	248	hypothetical protein
ATN84_02105	10,546	5	213	molecular chaperone GroES
ATN84_08900	14,093	9	195	glutaredoxin
ATN84_08230	20,761	4	192	ribosome recycling factor
ATN84_21155	14,202	3	161	hypothetical protein
ATN84_02900	22,651	6	155	hypothetical protein
ATN84_02440	27,160	5	149	glycosyl transferase
ATN84_17995	33,343	2	144	hypothetical protein
ATN84_03240	12,241	6	142	hypothetical protein
ATN84_14370	15,404	5	142	pseudo azurin
ATN84_12620	11,447	2	134	hypothetical protein

### Bioinformatics analysis of PAD-1

The gene ATN84_21655 (designated *bla*_*PAD*-*1*_) has an open reading frame (ORF) encoding a 297-amino-acid protein. A Conserved Domain Database search indicated that PAD-1 has two domains: a penicillin-binding protein transpeptidase domain (cl21491) at amino acids 44–291 and a β-lactamase class A (COG2367) domain at amino acids 9–292. This suggests that PAD-1 is a class A serine β-lactamase^5^.

A SWISS-MODEL analysis revealed that the class A carbapenemase KPC-2 in the database has a three-dimensional (3D) structure matching that of PAD-1(GQME value 0.67, QMEAN value −1.61), which is highly significant in carbapenem resistance, especially in clinical Enterobacteriaceae isolates^11^. Interestingly, PAD-1 also shares a similar 3D structure with an artificial class A β-lactamase, GNCA (GQME value 0.77, QMEAN value −0.13), which is the last common ancestor of the class A β-lactamases of the Gram-negative bacteria, predicted in a phylogenetic analysis^12^. Bayesian divergence estimates indicated that the ancestral GNCA gene was present on Earth about 2 billion years ago^12^.

**Figure 2:**
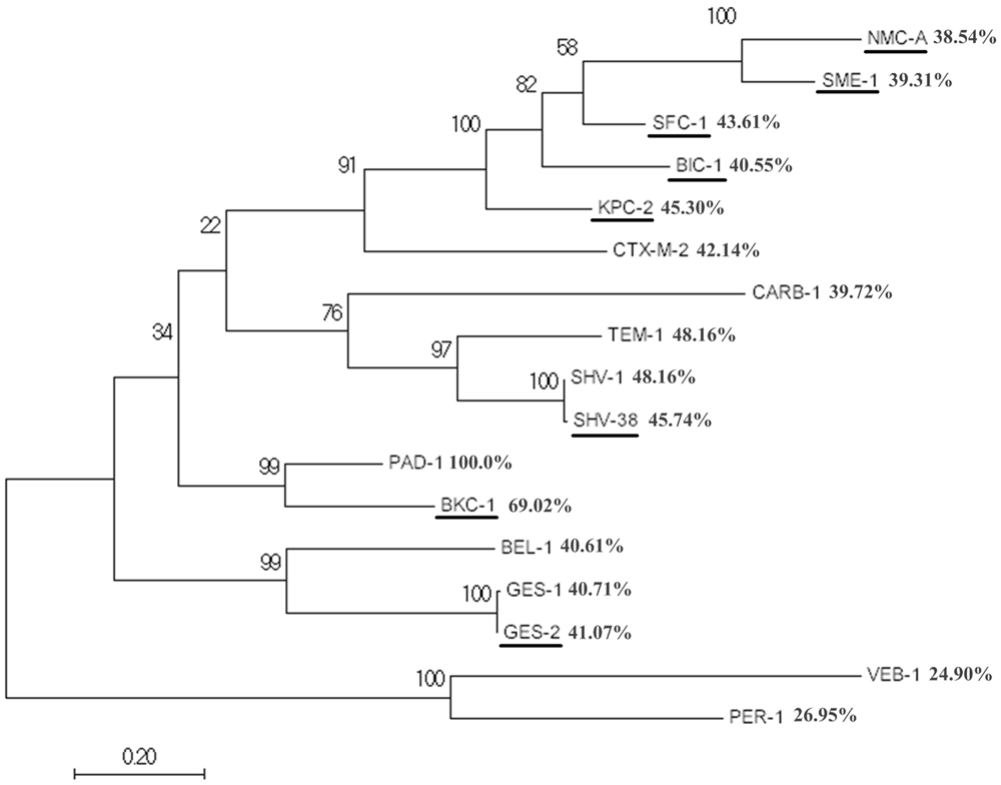
Molecular phylogenetic analysis by Maximum Likelihood method for PAD-1 and other class A β-lactamases. The evolutionary distances were computed using the Poisson correction method and are in the units of the number of amino acid substitutions per site. Numbers on the branches are bootstrap values. Underlined entries are reported carbapenemases. The amino acid homology values are given beside the lactamase names. Accession numbers for the 16 class A β-lactamases: SHV-1 (AKO62422.1), TEM-1 (AIL24699.1), CTX-M-2 (APD70461.1), CARB-1 (WP_063857835.1), GES-1 (AAF27723.1), VEB-1 (ACZ02434.2), PER-1 (ABC68520.1), BEL-1 (5EUA_B), SHV-38 (ACG58890.1), BKC-1 (AKD43328.1), KPC-2 (AJR19467.1), BIC-1 (WP_063857833.1), SFC-1 (AY354402.1), NMC-A (Z21956.1), SME-1 (CAA82281.1), and GES-2 (AAM08182.1).

**Figure 3:**
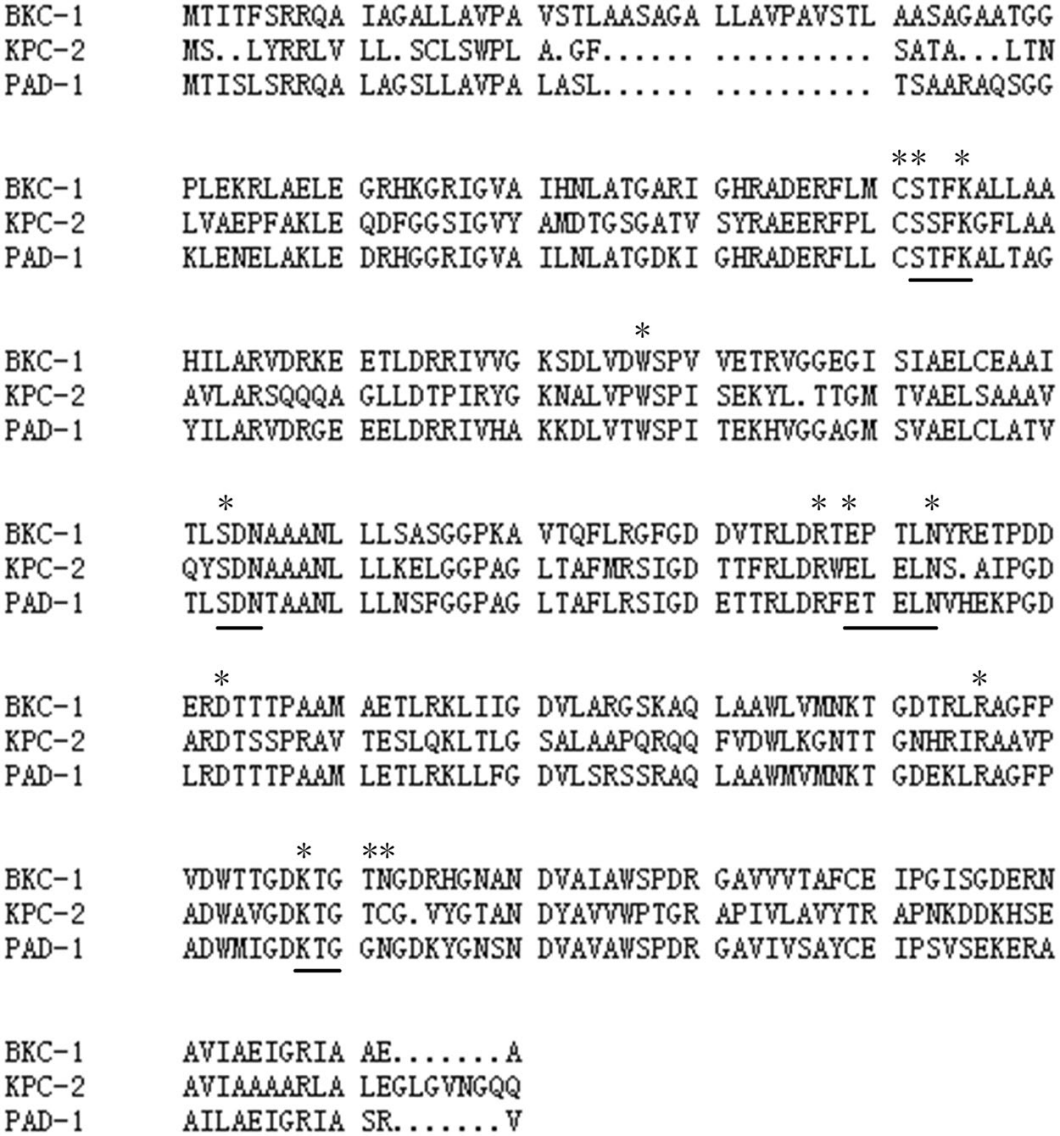
Amino acid alignment of PAD-1, BKC-1, and KPC-2. Conserved motifs of the class A serine β-lactamases are underlined. Asterisks mark residues considered important for class A carbapenemase activity.

Figure 2 shows a phylogenetic tree of PAD-1 and 16 other class A β-lactamases. BKC-1 is the closest relative of PAD-1 (69% amino acid identity). BKC-1 is a plasmid-encoded carbapenemase from a Brazilian clinical *K*. *pneumonia* isolate^13^. An amino acid sequence alignment of PAD-1, BKC-1, and KPC-2 (Fig. 3) shows that PAD-1 contains the four conserved structural elements of the class A serine carbapenemases: the motif 70-SXXK-73, where 70-S is the active serine of carbapenemase; the 130-SDN-133 loop; the single amino acid residue 166-E; and the motif 234-KTG-236^3, 14, 15^. PAD-1 also contains nearly all the reportedly important residues for class A carbapenemase activity (C69, S70, K73, H105, S130, R164, E166, N170, D179, R220, K234, S237, and C238), and only S237 and C238 are not conserved in PAD-1^3, 16–18^. Thus, the *in silico* bioinformatics analysis suggested that PAD-1 is a novel class A carbapenemase.

### *In vitro* susceptibility tests

To investigate the *in vitro* susceptibility of BL21 (DE3) strain carrying plasmid pET28a-*bla*_*PAD*-*1*_, we determined its MIC values against various β-lactams together with A-3-E^T^ and BL21 (DE3) carrying pET28a. As shown in Table 2, A-3-E^T^ strain exhibited high-level β-lactam resistance to most tested antibiotics, except for cephradine, cefoxitin and carbapenem. The BL21 (DE3) strain harboring pET28a-*bla*_*PAD*-*1*_ showed similar resistance spectrums to A-3-E^T^ strain, while BL21 (DE3) harboring pET28a is sensitive to all tested antibiotics. Our results suggested that PAD-1 was responsible for the β-lactam resistance detected in A-3-E^T^ strain. Interestingly, the MIC value of BL21 (DE3) harboring pET28a-*bla*_*PAD*-*1*_ for meropenem is four times higher than A-3-E^T^ strain (1 μg/ml vs 0.25 μg/ml). If induced with 0.1 mM IPTG, the MIC value of BL21 (DE3) harboring pET28a-*bla*_*PAD*-*1*_ for meropenem is even higher (8 μg/ml), which implies the expression level of PAD-1 is vital for the resistance profile against carbapenems in the host strain.

**Table 2:** MIC values of various β-lactams for A-3-E^T^ strain and *E*. *coli* BL21 (DE3) carrying *bla*_*PAD*-*1*_.

Antibiotics	MIC value (μg/ml)		
	A-3-E^T^	BL21 (DE3)+pET28a-*bla*_*PAD*-*1*_	BL21 (DE3)+pET28a
Ampicillin	>256	>256	<2
Piperacillin	256	128	<4
Ampicillin/Sulbactam	>256	>256	<2
Cefazolin	256	128	<4
Cephradine	4	16	<0.25
Cefoxitin	0.5	<0.25	<0.25
Cefuroxime	>128	>128	<1
Ceftazidime	>128	>128	<1
Ceftriaxone	>128	>128	<1
Cefepime	>128	>128	<1
Aztreonam	>128	>128	<1
Meropenem	0.25	1	<0.25
Meropenem + 0.1 mM IPTG	0.25	8	<0.25

### Effects of antibiotics on the transcription of *bla*_*PAD*-*1*_

**Figure 4:**
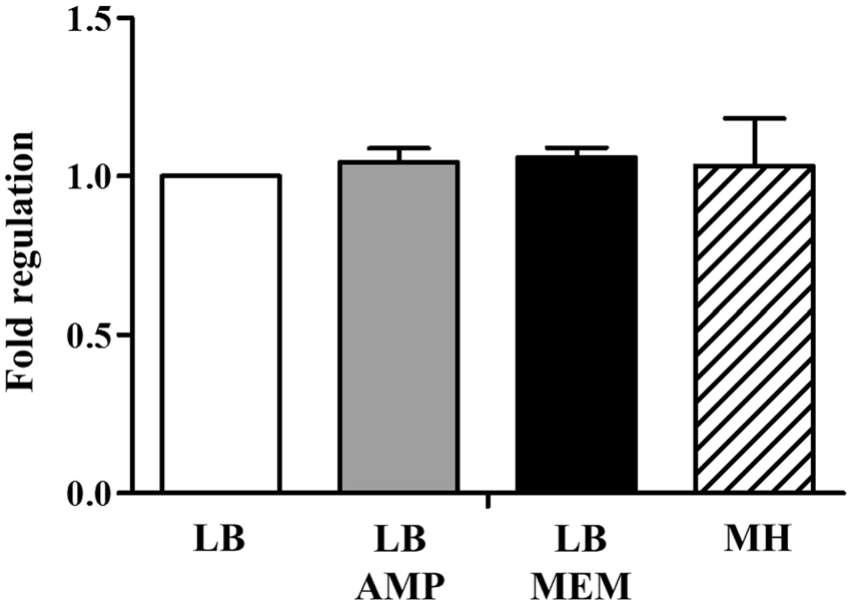
qRT-PCR for *bla*_*PAD*-*1*_ in A-3-E^T^. The transcription levels of *bla*_*PAD*-*1*_ in A-3-E^T^ strain grwon in medium with and without antibiotics (AMP: ampicillin 100 μg/ml, MEM: meropenem 32 μg/ml).

To determine whether the presence of antibiotics will influence the transcription of *bla*_*PAD*-*1*_, we measured mRNA level of *bla*_*PAD*-*1*_ in A-3-E^T^ strain grown in LB medium with or without ampicillin and meropenem by quantitative RT-PCR. As shown in Fig. 4, the expression levels of *bla*_*PAD*-*1*_ in A-3-E^T^ strain with antibiotic pressure are almost identical to that of in normal LB and MH medium. This immediately suggested that the expression PAD-1 is unlikely induced by antibiotics.

### Enzymatic kinetic parameters of PAD-1

The soluble expression and purification of PAD-1 was confirmed with sodium dodecyl sulfate-polyacrylamide gel electrophoreses (SDS-PAGE), and it was then subjected to enzyme kinetic assays. As shown in Table 3, PAD-1 hydrolysed penicillin, cephalosporins, carbapenems, and monobactams *in vitro*, but not cefoxitin. This is similar to BKC-1, which also does not hydrolyse cefoxitin^13^.

**Table 3:** Kinetic parameters of PAD-1, BKC-1, and KPC-2 against various β-lactam substrates.

Substrate	PAD-1			BKC-1			KPC-2		
	*K*_*m*_(μM)	*k*_*cat*_(s^−1^)	*k*_*cat*_/*K*_*m*_ (mM^−1^s^−1^)	*K*_*m*_(μM)	*k*_*cat*_(s^−1^)	*k*_*cat*_/*K*_*m*_ (mM^−1^s^−1^)	*K*_*m*_(μM)	*k*_*cat*_(s^−1^)	*k*_*cat*_/*K*_*m*_ (mM^−1^s^−1^)
Oxacillin	383.96	58.70	152.89	267.30	14,306.60	53,522.60	NA	NA	NA
Cefazolin	89.16	22.60	253.48	NA	NA	NA	16	44	2750
Cefalotin	91.59	15.20	165.96	NA	NA	NA	141	106	757.14
Cephradine	347.9	31.75	91.26	NA	NA	NA	NA	NA	NA
Cefoxitin	ND	ND	ND	ND	ND	ND	180	0.31	1.72
Ceftazidime	110.04	12.14	110.32	92.90	0.11	1.21	ND	ND	ND
Cefoperazone	47.80	15.08	315.48	NA	NA	NA	NA	NA	NA
Ceftriaxone	90.84	16.27	179.11	127.80	4.32	33.84	NA	NA	NA
Cefotaxime	137.08	20.06	146.34	223.90	0.46	2.08	174	66	379.31
Cefepime	108.40	10.4	95.94	174.30	1.69	9.69	NA	NA	NA
Meropenem	389.69	24.24	62.20	1.51	0.0034	2.25	15	4	266.67
Aztreonam	276.08	39.03	141.37	1,200.70	2.22	1.85	35	3	85.71

ND, no measurable hydrolysis detected; NA, data not available; data for BKC-1 from reference^13^; data for KPC-2 from references^11, 19^.

PAD-1 showed high affinity for cephalosporins (low *K*_*m*_ values), except cephradine, whose *K*_*m*_ value (347.9 μM) was three-fold higher than that of the other cephalosporins, implying that PAD-1 hydrolyses cephradine least efficiently (low *k*_*cat*_/*K*_*m*_ value). The *K*_*m*_ values of PAD-1 for oxacillin (383.96 μM) and meropenem (389.69 μM) suggested that it has less affinity for these antibiotics to this enzyme. According to the *k*_*cat*_/*K*_*m*_ values, cefoperazone (315.48 mM^−1^s^−1^) is the best substrate for PAD-1 and meropenem ranks last (62.20 mM^−1^s^−1^), although the difference is not significant.

Table 3 lists the published kinetic data for BKC-1 and KPC-2. BKC-1 is very efficient in the hydrolysis of oxacillin, with a *k*_*cat*_/*K*_*m*_ value 300 times higher than that of PAD-1, whereas the *k*_*cat*_/*K*_*m*_ values for other similar β-lactams are all lower than that of PAD-1^13^. Unlike PAD-1 and BKC-1, KPC-2 hydrolyses cefoxitin, and it seems to hydrolyse meropenem and cefalotin more efficiently than PAD-1^11, 19^.

## Discussion

In response to the lethal selection pressures of antibiotics, pathogenic bacteria have developed various mechanisms to fight back, including impermeable barriers, multidrug-resistant efflux pumps, resistance mutations, the inactivation of the antibiotics, etc.^20^. Under the selection of antibiotics, spontaneous mutations (such as the GyrA83 mutation conferring fluoroquinolone resistance) are fixed in the clinical bacterial populations in a very short time^21^. In contrast, more delicate mechanisms, such as antibiotic-hydrolysing enzymes and efflux mechanisms, do not evolve quickly, but must be acquired from other sources via horizontal gene transfer^22^. However, the reservoirs of antibiotic-resistance determinants have been poorly understood for a long time^23^.

Recently, metagenomic analyses identified diverse genes encoding resistance to β-lactam, tetracycline, and glycopeptide antibiotics in 30,000-year-old Beringian permafrost sediments, which are far more ancient than the very first human antibiotic discovered^24^. Soil microbiomes have been shown to be very important evolutionary origins of ancient antibiotic-resistance genes, and are vast reservoirs of novel antibiotic-resistance genes, which are exchanged with clinical pathogens^25, 26^.

However, the origins and diversity of antibiotic-resistance genes in soils throughout the world are still unclear. In previous studies, we isolated several β-lactam-resistant bacterial strains from soil samples from the Taklimakan Desert, the largest desert in the west of China, which is not affected by human activities such as farming or herding. One of these strains, A-3-E^T^, is extremely resistant to certain β-lactam antibiotics. For example, there was no significant difference between its growth curves in medium containing 8 mg/ml ampicillinor in normal medium, and A-3-E^T^ also grew well in medium containing 8 mg/ml carbenicillin, 1 mg/ml cefazolin, or 500 μg/ml cefotaxime. Based on systematic polyphasic taxonomic data, A-3-E^T^ was proposed as the type strain of the novel species *Paramesorhizobium desertii* ^9^. A nitrocefin assay of A-3-E^T^ suggested that β-lactamase contributes to its β-lactam-resistance phenotype^9^.

To investigate the β-lactamase (s) in A-3-E^T^, we analysed its genome^10^. Among the 4946 annotated genes, we identified 26 genes encoding potential β-lactamases or proteins containing β-lactamase-like domains, using a bioinformatics analysis. When we performed a BLAST search for these proteins against public databases, all the top hits were *in silico*-annotated β-lactamases, although with no experimental functional support. We randomly selected six genes of the 26 candidates, amplified their intact ORFs, and cloned them into expression vectors. Unfortunately, we failed to detect β-lactamase activity in any of the six proteins.

We then used a novel strategy to identify the potential β-lactamase (s). We first confirmed the existence of active β-lactamase (carbapenemase) in the freeze-thawed supernatant of an A-3-E^T^ culture using a modified Hodge assay with imipenem and meropenem (Fig. 1). We then performed a shotgun proteomic analysis of the supernatant, which immediately identified ATN84_21655 (*bla*_*PAD*-*1*_) as a candidate carbapenemase. In previous studies, to identify novel β-lactamases with low sequence similarity to known β-lactamases, researchers performed tedious time-consuming procedures, including purifying the lactamase from crude extracts, estimating the isoelectric point (pI) with an isoelectric-focusing-nitrocefin assay, fragment cloning and mapping, etc.^27, 28^. The strategy developed in this study is straight forward and efficient, and should have great utility in identifying novel β-lactamases in other bacteria.

Of the 12 β-lactam antibiotics tested in the enzyme kinetic assays, cefoxitin was the only one not hydrolysed by PAD-1, which is consistent with the cefoxitin-sensitive phenotype of A-3-E^T^. In contrast, A-3-E^T^ is sensitive to cephradine (MIC, 4 μg/ml), whereas purified PAD-1 hydrolysed cephradine *in vitro* with strong enzyme activity (*k*_*cat*_/*K*_*m*_ 91.26 mM^−1^s^−1^). Interestingly, this was also the case for meropenem, in that the results were positive for a modified Hodge assay of the A-3-E^T^ supernatant on meropenem MH plates, and an enzyme kinetic assay indicated a high *k*_*cat*_/*K*_*m*_ value (62.20 mM^−1^s^−1^) for meropenem with purified PAD-1. Classical MIC tests defined A-3-E^T^ as carbapenem-sensitive (imipenem 0.38 μg/ml, meropenem 0.25 μg/ml), while it grows well in Luria–Bertani (LB) broth containing 32 μg/ml imipenem or meropenem^9^.

One possible explanation is that the conflict results from MIC assays and LB broth is caused by certain inducible carbapenemases. If induced with 0.1 mM IPTG, the MIC value of BL21 (DE3) harboring pET28a-*bla*_*PAD*-*1*_ is 8 times higher than that of BL21(DE3) not induced by IPTG (8 μg/ml vs 1 μg/ml, Table 2). As shown in Table 3, the *k*_*cat*_/*K*_*m*_ of PAD-1 in the meropenem assay was nearly 30 times higher than that of BKC-1 (62.20 mM^−1^s^−1^ vs 2.25 mM^−1^s^−1^, respectively), whereas the MIC of meropenem for A-3-E^T^ was much lower than that of a clinical *K*. *pneumonia* isolate containing plasmid-borne *bla*_*BKC*-*1*_ (0.25 μg/ml vs 32 μg/ml)^13^. These results implies that the protein level of PAD-1 do infect the MIC values.

However, our qRT-PCR assays showed that the transcription level of PAD-1 in A-3-E^T^ is not affected by ampicillin (100 μg/ml) or meropenem (32 μg/ml). Figure 4 revealed that neither ampicillin/meropenem nor components in LB broth will induce the expression of PAD-1. Notably, the supernatant used in the modified Hodge assay was concentrated and the enzyme kinetic assays were performed with purified PAD-1. In standard MIC assays and both experiments confirm its activity to hydrolyse carbapenems. In microdilution broth MIC assay, A-3-E^T^ was cultured in MH medium for 24 hours without shaking, while it is able to grow in LB or MH broth containing meropenem with shaking. It is known that lots of factors (inoculum size, type medium, incubation time, etc.) can influence MIC values^29–31^. A most likely explanation is that the low level constitutive expression PAD-1 (chromosome- encoded) will lead to low values in MIC assays (MH medium, without shaking), while shaking culture in LB medium might favour the growth of A-3-E^T^ and the accumulation of PAD-1 help it to resist high concentration of meropenem.

PAD-1 is a class A serine carbapenemase with an amino acid sequence similar to those of clinically identified enzymes, such as BKC-1 (66%) and KPC-2 (47%)^11, 13^. Unlike KPC-2, neither PAD-1 nor BKC-1 hydrolyses cefoxitin. Although these three proteins share several common motifs and key amino acids essential for class A serine carbapenemases (70-SXXK-73, 130-SDN-133, 234-KTG-236, etc.)^3, 14^, they also contain several variant amino acid. It has been reported that residues C69 and C238 of KPC-2 form a disulfide bond^32^, whereas amino acid 238 in PAD-1 and BKC-1 is asparagine (N). This missing disulfide bond may contribute to the differences in the hydrolytic efficiency for certain β-lactams between PAD-1 and KPC-2. In another class A serine carbapenemase, SME-1, the disruption of the C69–C238 disulfide bond causes the loss of hydrolytic activity against imipenem and cefotaxime^33^. The relationship between the amino acid diversity and the enzymatic activities of the carbapenemases warrants further study.

In the genomic analysis, we identified no plasmid in the genome of A-3-E^T^. The G+C content of the *bla*_*PAD*-*1*_ gene (63.87%) is similar to that of the A-3-E^T^ strain (60.93%) and there is no identifiable transfer structure near *bla*_*PAD*-*1*_^10^. Because there are no mobile elements neighbouring *bla*_*PAD*-*1*_, it would be difficult for *bla*_*PAD*-*1*_ to move into other bacteria. Therefore, we assume that *bla*_*PAD*-*1*_ is inherent to this *P*. *desertii* strain rather than acquired from other bacterial species, or that the acquisition event occurred sufficiently long ago for any evidence of chromosomal recombination to have been smoothed away. It is noteworthy that the modelled 3D structure of PAD-1 is similar to that of GNCA (Global Model Quality Estimation, GMQE 0.73). GNCA is a laboratory-resurrected class A β-lactamase based on a comprehensive phylogenetic analysis, which is considered to be the last common ancestor of the class A β-lactamases of various Gram-negative bacteria. The divergence time of GNCA and modern class A β-lactamase is estimated to have been 2 billion years ago (Precambrian). Despite its extensive sequence differences from modern enzymes (∼100 amino acid differences), the catalytic efficiency of GNCA for various antibiotics is similar to theirs^12^.

In conclusion,we have identified a novel chromosome-encoded class A carbapenemase, PAD-1, in *P*. *desertii* strain A-3-E^T^ with unusual β-lactam-resistance characteristics. Because this strain was isolated from soil samples collected in the Taklimakan Desert, a natural environment unaffected by human activities, PAD-1 should extend our understanding of the diversity and evolutionary scenarios of environmental carbapenemases.

## Materials and Methods

### Bacterial strains and plasmids

The A-3-E^T^ strain used in this study was isolated from Taklimakan Desert, China, and proposed as the type strain for *P*. *desertii*, the type species of the novel genus *Paramesorhizobium* ^9^. Plasmid pET28a and *E*. *coli* BL21(DE3) were used to express the proteins *in vitro*. Both strain A-3-E^T^ and *E*. *coli* were cultured in LB medium (1% tryptone, 0.5% yeast extract, 1% NaCl) at 37 °C. A carbapenem-sensitive *E*. *coli* (American Type Culture Collection [ATCC]25922) was used in the modified Hodge assay to visualize the zones of antibiotics inhibition.

### Whole-genome sequencing

To detect the antibiotic resistance gene, we sequenced the whole genome of strain A-3-E^T^ with the Illumina HiSeq. 2000 platform using a paired-end strategy. The putative resistance genes were predicted with GeneMarkS and then annotated by searching public databases (KEGG, COG, and NR) with BLAST^34, 35^, as has been previously described in detail^10^.

### Detection of carbapenemase activity

The A-3-E^T^ strain was cultured in LB medium to stationary phase, and 20 ml bacteria were harvested and resuspended in 1 ml of distilled water. The bacterial culture was then frozen at −70 °C for 1.5 h, thawed at room temperature, and the freeze–thaw procedure was repeated five times to lyse the bacteria. After centrifugation, the supernatant was collected and sterilized with 0.22 μM filters (Millipore Inc., Massachusetts, USA), then a cloverleaf test (modified Hodge test) was used to detect carbapenemase production^36^. Briefly, *E*. *coli* ATCC 25922 was plated onto MH agar and allowed to dry for 5 min. Meropenem and imipenem discs (Biomerieux Inc., France) were placed in the centres of the agar plates and three grooves were dug around the discs. The A-3-E^T^ supernatant (200 μl) was added to one groove, 200 μl of distilled water was added to another as the control, and the last groove was left empty. The MH agar plates were incubated at 37 °C for 24 h.

### Proteomic analysis

To identify the carbapenemase in the supernatant, we used a brief proteomic analysis. The supernatant was mixed with loading buffer in a 100 °C water-bath for 10 min, and separated with 12% (w/v) SDS-PAGE. The gel was stained with Coomassie Brilliant Blue R-250. The visible bands in the gel were digested with trypsin and sent for proteomic analysis. All the digested peptides were analysed with a SYNAPT G2 Mass Spectrometer (Waters Inc., USA). The results were processed with PLGS 2.3 and the resulting peaklists were identified with the annotated genome of strain A-3-E^T^, as previously described^37^.

### Bioinformatics analysis of PAD-1

The amino acid sequence of PAD-1 was compared with the Conserved Domains Database at the National Center for Biotechnology Information^38^. The 3D protein structure of PAD-1 was then predicted at the SWISS-MODEL server^39^. An amino-acid-based phylogenetic tree containing 16 typical class A β-lactamases (KPC-2, BKC-1, BIC-1, TEM-1, CTX-M-2, etc.) was constructed with MEGA 6.0 by using the maximum likelihood algorithm^40^.

### Expression and purification of carbapenemase PAD-1

To purify PAD-1, we cloned the *bla*_*PAD*-*1*_ gene into the expression vector pET28a, under the control of the T7 promoter^41^. The intact *bla*_*PAD*-*1*_ gene was amplified from the DNA of strain A-3-E^T^ with primers 5088_F (5′-CTAGCTAGCATGACGATATCCCTTTC-3′) and 5088_R (5′-CCGGAATTCTTAGACCCGCGAAGC-3′), containing *Nhe*I and *Eco*RI restriction sites (underlined), respectively. The PCR product was purified with the QIAquick PCR Purification Kit (Qiagen Inc., USA). Both the purified PCR product and the expression vector pET28a were digested with restriction endonuclease *Nhe*I and *Eco*RI (New England Biolab Inc.) and ligated with T4 DNA ligase. The recombinant plasmid pET28a-*bla*_*PAD*-*1*_ was then introduced into *E*. *coli* BL21(DE3).

*Escherichia coli* BL21(DE3) carrying the plasmid pET28a-*bla*_*PAD*-*1*_ was grown in LB medium containing kanamycin (50 μg/ml) at 37 °C to an optical density at 620 nm (OD_620_) of 0.6. Isopropyl β-d-thiogalactopyranoside (IPTG; final concentration 0.1 mM) was added and incubated at 20 °C with shaking at 100 rpm for 5 h. The bacterial cells were harvested by centrifugation, resuspended in 10 ml of lysis buffer (300 mM NaCl, 50 mM NaH_2_PO_3_, 10 mM imidazole, pH 8.5), and then disrupted by sonication. The lysate was centrifuged and the supernatant was collected. The protein was isolated from the supernatant with a flow column containing Ni-NTA Agarose (Qiagen Inc., Germany). The purity of the protein was estimated with SDS-PAGE and the concentration of the protein was measured with a commercial BCA assay kit (Thermo Scientific, USA)^42, 43^. The purified PAD-1 protein was stored at −20 °C.

### *In vitro* susceptibility tests

The MIC values of A-3-E^T^ strain, *E*. *coli* BL21 (DE3) harboring pET28a and pET28a-*bla*_*PAD*-*1*_ were determined by the broth dilution method using Mueller-Hinton (MH) broth. All the bacterial isolates were grown on the MH agar plates at 37 °C overnight to reach stationary phase and the colonies were resuspended in MH broth to demanded concentration. Then the cell cultures were inoculated into the cell plates containing MH broth with a range of β-lactam concentrations. The MIC values were determined after 24 h incubation at 37 °C.

### Quantitative RT-PCR of *bla*_*PAD*-*1*_

Quantitative RT-PCR (qRT-PCR) was performed to compare the expression levels of the *bla*_*PAD*-*1*_ transcript in the A-3-E^T^ strain with and witout antibiotics. The A-3-E^T^ strains were firstly grown in LB medium (normal, ampicillin 100 μg/ml, meropenem 32 μg/ml) and MH medium, and all the RNA samples were extracted with the Pure Link^TM^ RNA Mini Kit (Invitrogen, Carlsbad, CA, USA). The cDNA was synthesized from the RNA samples with the Thermo Scrip RT-PCR System (Invitrogen, Carlsbad, CA, USA). The qRT-PCR reactions were performed in duplicate with 20 ng cDNA template on the LightCycler^®^ 480 II Real-Time PCR System (Roche, Burgess Hill, UK) using SYBR^®^ Premix Ex *Taq*™ II (Takara, Japan). Three biological replicates were performed for each samples. 16S rRNA was used as an internal standard (16S-F: 5′-GGGAGTACGGTCGCAAGA-3′, 16S-R: 5′-GGATGTCAAGGGCTGGTAA-3′) and the *bla*_*PAD*-*1*_ was amplified with primers PAD-1_F: 5′-TGACCCTGAGCGACAACACC-3′, PAD-1_R:5′-CACCGATGGAGCGCAAAA-3′.

### Enzyme kinetic assays of PAD-1

The enzyme kinetic parameters (*k*_*cat*_ and *K*_*m*_) of purified PAD-1 were assayed spectrophotometrically in sterile phosphate-buffered saline (50 mM, pH 7.0) at 37 °C. The purified carbapenemase PAD-1 was added to 80 μl solutions of various antibiotics and the initial hydrolysis rates were determined with a SpectraMax M2 Microplate Reader (Molecular Devices., USA), as previously reported^11^. The absorption wavelengths used to measure the kinetic parameters for the different antibiotics were determined by spectrum scanning: oxacillin, 220 nm; cefazolin, 265 nm; cefalotin, 265 nm; cephradine, 265 nm; cefoxitin, 235 nm; ceftazidime, 255 nm; cefoperazone, 266 nm; ceftriaxone, 255 nm; cefotaxime, 259 nm; cefepime, 265 nm; meropenem, 290 nm; and aztreonam, 310 nm. All antibiotics were purchased from the National Institutes for Food and Drug Control, China. The values of the kinetic parameters (*k*_*cat*_ and *K*_*m*_) were estimated with Lineweaver-Burk linearization of the Michaelis-Menten equation^13^.
